# Three-monthly gonadotropin-releasing hormone agonist for ovarian function suppression in premenopausal breast cancer: a systematic review and meta-analysis

**DOI:** 10.1007/s10549-026-07960-2

**Published:** 2026-06-12

**Authors:** Caio Dabbous de Liz, Pedro C. Abrahão Reis, Ellen R. Blanchard-Cavagis, Isabela C. Diniz, Heloísa Carneiro Brito, Filipe Luis Vasconcelos Visani

**Affiliations:** 1 Hospital Paraná, Rede Américas, Maringá, Paraná Brazil; 2 Universidade Federal Do Rio de Janeiro (UFRJ), Rio de Janeiro, Rio de Janeiro Brazil; 3 Université Paris Cité, Paris, France; 4 Universidade Estadual de Campinas (UNICAMP), Campinas, São Paulo, Brazil; 5 Universidade Federal da Paraíba (UFPB), João Pessoa, Paraíba Brazil; 6 Oncoclínicas, Salvador, Bahia Brazil

**Keywords:** Breast cancer, Endocrine therapy, Gonadotropin release-hormone agonist, Ovarian function suppression, Premenopausal

## Abstract

**Purpose:**

Ovarian function suppression (OFS) with gonadotropin-releasing hormone agonists (GnRHa) improves outcomes in premenopausal women with hormone receptor–positive (HR +) breast cancer (BC), most commonly using monthly administration. The potential equivalence of 3-monthly (3 M) versus monthly (1 M) schedules has been suggested but remains uncertain.

**Methods:**

We performed a systematic review and meta-analysis of interventional studies comparing 3 M and 1 M GnRHa regimens in premenopausal women with HR + BC receiving endocrine therapy. Outcomes included ovarian escape (OE), mean estradiol (E2) levels, disease-free survival (DFS), progression-free survival (PFS), and adverse events. Subgroup analyses were performed by estradiol assay type, and a sensitivity analysis restricted to RCTs.

**Results:**

Fifteen studies comprising 4,324 patients were included, of whom 2,098 (48%) received a 3 M regimen. The pooled OE was 10% and showed no significant difference between schedules (RR 0.86; 95% CI 0.60–1.22; p = 0.39). Ultrasensitive assays yielded higher OE detection (31%) without inter-regimen differences (RR 0.90; 95% CI 0.38–2.14). Mean E2 concentrations at 12 weeks were similar (MD 1.36 pg/mL; 95% CI –3.66 to 6.38), with consistent findings in RCT-only analyses (MD –1.77 pg/mL; 95% CI –5.71 to 2.17). DFS (RR 1.02; 95% CI 0.67–1.56) and PFS (RR 0.86; 95% CI 0.72–1.04) were also comparable. No clinically relevant differences were observed in reported safety outcomes.

**Conclusion:**

3 M GnRHa achieved OFS efficacy comparable to 1 M regimens, with no meaningful differences in survival or safety. The findings support 3 M GnRHa as a valid and practical alternative for OFS in appropriately selected premenopausal women with HR + BC.

**Supplementary Information:**

The online version contains supplementary material available at 10.1007/s10549-026-07960-2.

## Introduction

Breast cancer (BC) remains the most commonly diagnosed malignancy and the leading cause of cancer-related mortality among women worldwide [1, 2]. In 2025 alone, an estimated 319,750 new BC cases occurred in the United States and 73,610 in Brazil, with more than 42,000 and 20,000 deaths, respectively [3, 4]. Although the overall burden of BC continues to rise in nearly all regions, important differences exist according to menopausal status. Premenopausal BC, in particular, imposes a disproportionately higher burden on low- and medium-human development index (HDI) countries, where both incidence and mortality rates are increasing more rapidly than in high-HDI settings [5], reflecting marked disparities in risk factors, screening access, and therapeutic resources. Additionally, this impact is especially pronounced among premenopausal women, in whom hormone receptor–positive (HR +) disease is common and therapeutic strategies must balance oncologic efficacy with the preservation of reproductive health and overall quality of life [5].

For more than a century, ovarian function suppression (OFS) has constituted a fundamental component of endocrine management for HR + BC [6]. Gonadotropin-releasing hormone agonists (GnRHa) provide reversible OFS by suppressing gonadotropin release and ovarian steroidogenesis [7]. In premenopausal women with HR + BC, GnRHa therapy has emerged as an effective, less invasive alternative to permanent surgical oophorectomy or ovarian irradiation, with comparable efficacy and the key advantage of potential fertility preservation [8–10].

In the contemporary era of optimized endocrine therapy (ET), landmark trials have demonstrated that the addition of OFS to tamoxifen or aromatase inhibitors (AIs) significantly improves outcomes among pre- and perimenopausal women with high-risk HR + disease [11–14]. Consequently, international guidelines endorse the use of GnRHa in combination with tamoxifen or AIs as standard therapy for this population [15–17]. Historically, monthly depot formulations have been the most extensively investigated and widely adopted GnRHa regimen. Although effective, monthly administration may be associated with greater treatment burden, increased toxicity, and adherence challenges that could compromise long-term endocrine control. In young women who must balance oncologic control with fertility considerations, treatment burden, and quality of life, reducing injection frequency may represent a clinically meaningful advantage [18–21]. In this contex, three-monthly (12-week) formulations were subsequently developed to improve treatment convenience and patient adherence while maintaining adequate ovarian suppression. Evidence from clinical studies suggests that extended-interval dosing may achieve comparable estradiol suppression and clinical efficacy [22, 23].

Nevertheless, although several studies have suggested possible equivalence between three-monthly (3 M) and monthly (1 M) GnRHa [24–32], and despite their incorporation into the NCCN Breast Cancer Guidelines [33] and approval by some regulatory agencies [34–36], the comparative effectiveness of 3 M versus 1 M GnRHa remains uncertain and is not yet an established standard. Given the absence of any recent comprehensive review on this topic, we conducted a systematic review and meta-analysis to evaluate the efficacy and safety of 3 M versus 1 M GnRHa regimens in premenopausal women with HR + BC receiving ET.

## Material and methods

This systematic review and meta-analysis was conducted in accordance with the Cochrane Handbook for Systematic Reviews of Interventions and reported in line with the Preferred Reporting Items for Systematic Reviews and Meta-Analyses (PRISMA) statement [37, 38]. The study protocol was prospectively registered in the PROSPERO database (CRD420251118803).

### Eligibility criteria

Studies were considered eligible if they met all the following criteria: (1) randomized controlled trials (RCTs) or non-randomized studies of intervention (NRSIs); (2) enrollment of premenopausal women with HR + BC undergoing reversible OFS with GnRHa; (3) reporting outcome data for both 3 M and 1 M regimens, irrespective of whether a direct comparison was performed; and (4) a minimum follow-up of 12 weeks. Eligible studies were further required to report at least one of the prespecified clinical outcomes of interest.

The 12-week minimum follow-up threshold was defined to ensure adequate assessment of OFS and associated biochemical endpoints, as this interval represents the earliest clinically meaningful time point for hormonal evaluation under GnRHa therapy. Studies reporting hormonal outcomes at 12 weeks were therefore considered eligible, even in the absence of longer-term survival data. Survival outcomes, when available, were extracted and analyzed according to the follow-up durations specified in the individual studies, without imposing additional time-based restrictions.

Studies were excluded if they met any of the following criteria: (1) investigation of GnRHa exclusively for fertility preservation; or (2) inclusion of overlapping populations from the same research group, unless distinct outcomes or follow-up periods were reported; in such cases, the most comprehensive dataset was preferentially included.

Fertility preservation protocols were excluded because this indication differs biologically and clinically from continuous OFS administered as part of ET for HR + BC. Fertility preservation typically involves temporary GnRHa administration initiated in proximity to chemotherapy, with distinct timing, duration, and therapeutic objectives. Inclusion of such studies would have introduced clinical and mechanistic heterogeneity into the present analysis.

### Search strategy and data extraction

We systematically searched PubMed, Embase, and Cochrane Library from database inception through July 2025, using the following search terms: ‘GnRH agonists’, ‘LHRH agonists’, ‘goserelin’, ‘leuprorelin’, ‘triptorelin’, ‘3-monthly’, ‘12 weeks’, ‘monthly’, ‘4 weeks’, and ‘breast cancer’. The search was conducted without language restrictions. The full search strategy, applied consistently across databases, is detailed in Supplementary Table 1. Additionally, abstracts presented at the American Society of Clinical Oncology (ASCO) Annual Meetings (2021–2025) were screened to identify potentially relevant unpublished studies, and the reference lists of all eligible articles were manually reviewed to capture additional publications.

Two authors (C.D.L. and F.L.V.V.) independently extracted the data using a predefined standardized form that included study characteristics, patient demographics, treatment regimens, and outcomes of interest. Discrepancies between reviewers were resolved through discussion and consensus.

### Endpoints and sub-analyses

Efficacy outcomes included: (1) estradiol suppression; (2) ovarian escape (OE); (3) mean serum concentrations of estradiol (E2), follicle-stimulating hormone (FSH), and luteinizing hormone (LH); (4) disease-free survival (DFS) and progression-free survival (PFS) events; and (5) all-cause mortality. Safety outcomes of interest were hot flashes, arthralgia, headache, and nausea, as reported in the original studies.

Estradiol suppression was defined as serum E2 concentrations below the menopausal threshold specified in each trial. OE was defined according to study-specific criteria and was considered present when at least one reported serum E2 measurement exceeded the menopausal threshold during GnRHa therapy. Mean serum E2, FSH, and LH concentrations were assessed at 12 weeks of follow-up. DFS events were defined as disease recurrence or death, and PFS events as disease progression or death.

Assay methodologies and cut-off thresholds used to define menopausal E2 levels varied across studies, as summarized in Table 1. To address this heterogeneity, subgroup analyses were prespecified according to estradiol assay type (conventional immunoassay vs. ultrasensitive assays). A sensitivity analysis restricted to RCTs was also planned for mean E2 levels to assess the robustness and consistency of findings across study designs.

**Table 1:** Baseline characteristics of included studies in the meta-analysis

Study year	Design	Median follow up; mo (range)	Experimental group—3 M	Patients	Age^d^, y	ABC, %	Prior CT, %	E2 assay	E2 cut-off for OFS, pg/mL
			Control group—1 M						
Aydiner 2013	Prospective	27 (9–66)	3 M—Leuprorelin + TMX	41	40	0	88	IA	30
			1 M—Goserelin + TMX	38	39	0	87		
Blotta 2023^a^	Retrospective	NA	3 M—Goserelin + AI	61	39	11	93	UA	2.72
			1 M—Goserelin + AI	27	39	18	77		
Boccardo 1999	RCT	NA	3 M group—Leuprorelin	27	42	11	70	IA	46
			1 M—Leuprorelin	23	41	4	78		
Burns 2021	Retrospective	NA	3 M—Leuprorelin/Goserelin + AI/TMX	8	42^e^	0	59^e^	UA	2.7^i^
			1 M—Leuprorelin/Goserelin + AI/TMX	38		0			
Chen 2024	Retrospective	NA	3 M—Leuprorelin + AI	84	43	57	95	IA	30
			1 M—Leuprorelin/Goserelin + AI	180	43^f^	45	87^ h^		
El Zawawy 2022^a^	Retrospective	21 (NA)	3 M—Goserelin + AI/TMX	45	37	29	NA	NA	NA
			1 M—Goserelin + AI/TMX	42	35	26	NA		
Kendzierski 2018	Retrospective	NA	3 M—Leuprorelin + AI	101	40	0	53	NA	40
			1 M—Leuprorelin + AI	100	39	0	53		
Lin 2024^b^	Retrospective	96 (NA)^j^	3 M—Leuprorelin/Goserelin + AI/TMX	475	40	0	91	IA	30
			1 M—Leuprorelin/Goserelin + AI/TMX	475	39	0	90		
Masuda 2011	RCT	22.5 (4.7–22.9)	3 M—Goserelin + TMX	86	43	0	0	IA	30
		22.5 (5.3–22.8)	1 M—Leuprorelin/Goserelin + TMX	84	44	0	0		
McCann 2025	Retrospective	36.3 (NA)	3 M—Goserelin ± AI/TMX	670	42	13	NA	NA	NA
		37.1 (NA)	1 M—Goserelin ± AI/TMX	700	41	15	NA		
NCT00322348 2010^c^	RCT	NA	3 M—Goserelin + TMX	49	42	100	NA	NA	NA
			1 M—Goserelin + TMX	49	43	100	NA		
Noguchi 2016	RCT	NA	3 M—Goserelin + TMX	109	41	100	27	IA	30
			1 M—Goserelin + TMX	113	42	100	24		
Sarantis 2024	RCT	NA	3 M—Leuprorelin/Triptorelin + AI/TMX	13	48	0	77	NA	30
			1 M—Triptorelin + AI/TMX	12	46	0	67		
Tesch 2024	Prospective	84 (12–156)	3 M—GnRHa + AI/TMX	34	NA^g^	14^e^	58^e^	UA	2.72
			1 M—GnRHa + AI/TMX	47	NA^g^				
Wang 2024^b^	Retrospective-prospective	NA	3 M—Goserelin + AI/TMX	295	NA	4	60	NA	NA
			1 M—Goserelin + AI/TMX	295	NA	3	60		

^a^conference abstracts

^b^overlapping study location and recruitment period

^c^reported only in the ClinicalTrials.gov registry

^d^mean or median

^e^all population

^f^value in leuprolide subgroup

^g^restricted to patients younger than 40 years

^h^statistical difference between 3 and 1 M groups

^i^21 pg/mL if on tamoxifen

3 M 3-Monthly regimen, 1 M Monthly regimen, ABC Advanced breast cancer, AI Aromatase inhibitor, CT Chemotherapy, E2 Estradiol, GnRHa Gonadotropin-releasing hormone agonist, IA Conventional immunoassay, NA Not available, NRSI Non-randomized study of intervention, OFS Ovarian function suppression, Mo months, RCT Randomized controlled trial, TMX Tamoxifen, UA Ultrasensitive assay

### Quality assessment

For RCTs, risk of bias was assessed using the Cochrane Risk of Bias 2 (RoB 2) tool, which classifies trials as having low risk, some concerns, or high risk of bias across specified domains [39]. NRSIs were appraised with the ROBINS-I tool, which is structured around signaling questions and domain-specific judgments and provides an overall classification of low, moderate, serious, or critical risk of bias [40]. All evaluations were conducted independently by two reviewers (C.D.L. and F.L.V.V.), with discrepancies resolved by consensus.

Potential publication bias was assessed by visual inspection of a funnel plot of effect estimates against study weights. As fewer than ten studies contributed to each outcome, formal statistical testing for small-study effects was not performed. For prespecified outcomes of interest, the certainty of the evidence was appraised using the Grading of Recommendations, Assessment, Development and Evaluation (GRADE) approach, considering study limitations, inconsistency, indirectness, imprecision, and publication bias [41].

### Statistical analysis

For binary endpoints, pooled risk ratios (RRs) with 95% confidence intervals (CIs) were calculated. For continuous variables, effect sizes were estimated as mean differences (MDs) with corresponding 95% CI. Between-study heterogeneity was assessed using Cochrane’s Q-test and I^2^ statistic, with a restricted maximum-likelihood estimator model. Heterogeneity was classified as low (I^2^ < 25%), moderate (25%–50%), or high (I^2^ > 50%). To account for anticipated clinical and methodological variability, pooled estimates were generated using the DerSimonian–Laird random-effects model [42]. All statistical analyses were conducted with Review Manager Web (RevMan Web, The Cochrane Collaboration).

## Results

### Study selection and characteristics

**Fig. 1:**
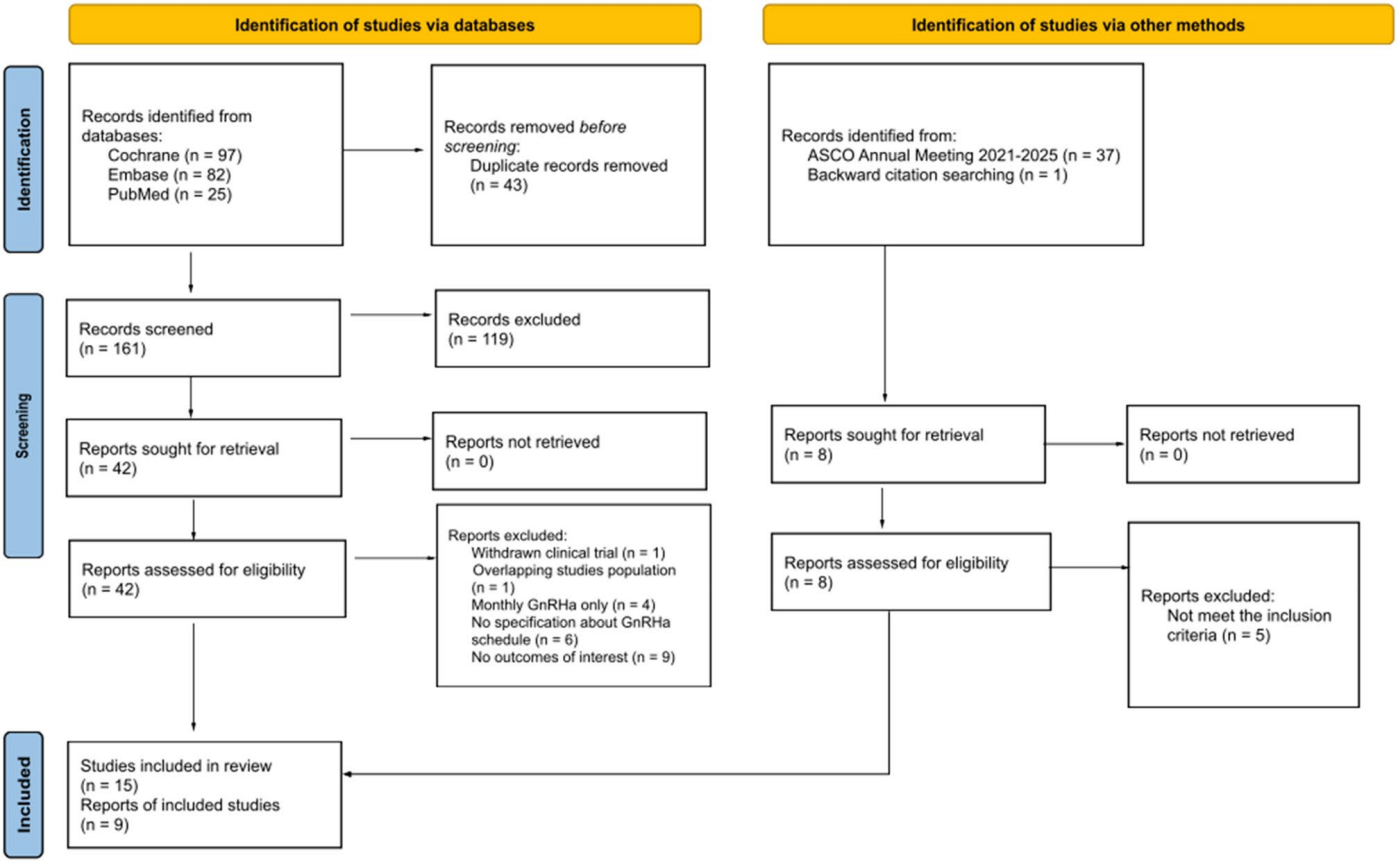
PRISMA flow diagram of study screening and selection

As outlined in the PRISMA flow diagram Fig. 1, the search yielded 242 records. After removal of duplicates and screening for eligibility, 50 full-text articles were assessed, of which 15 met eligibility criteria, encompassing 4,324 patients; 2,098 (48%) received 3 M GnRHa. The final dataset comprised five RCTs [25, 29, 31, 43, 44] and ten NRSIs [24, 26–28, 30, 32, 45–48]. All NRSIs were observational cohort studies, including two prospective cohorts, seven retrospective cohorts, and one retrospective–prospective cohort. Two conference abstracts [26, 45] and one RCT available exclusively through ClinicalTrials.gov [44], all of which provided sufficient data for analysis, were included. Two studies [28, 32] were identified as potentially involving overlapping cohorts based on recruitment period and study sites; in such cases, each contributed distinct outcomes, thereby preventing duplication of endpoints in the pooled analyses.

Baseline characteristics of the included studies are summarized in Table 1. The mean or median age of participants ranged from 36 to 47 years, with one trial restricted to patients younger than 40 years [48]. Two trials enrolled only patients with advanced BC, six focused solely on early stage disease, and the remaining studies included mixed populations comprising both early and advanced cases. Prior exposure to chemotherapy was heterogeneous, ranging from 0% to over 90%. Regarding concomitant ET, GnRHa were combined with AI in three studies and with tamoxifen in six, whereas several cohorts permitted either agent. In three studies, estradiol was measured with ultrasensitive assays (OFS defined as E2 < 2.7 pg/mL), whereas six studies used conventional immunoassays, applying cut-offs of < 30 pg/mL or < 46 pg/mL for OFS.

### Efficacy of OFS

**Fig. 2:**
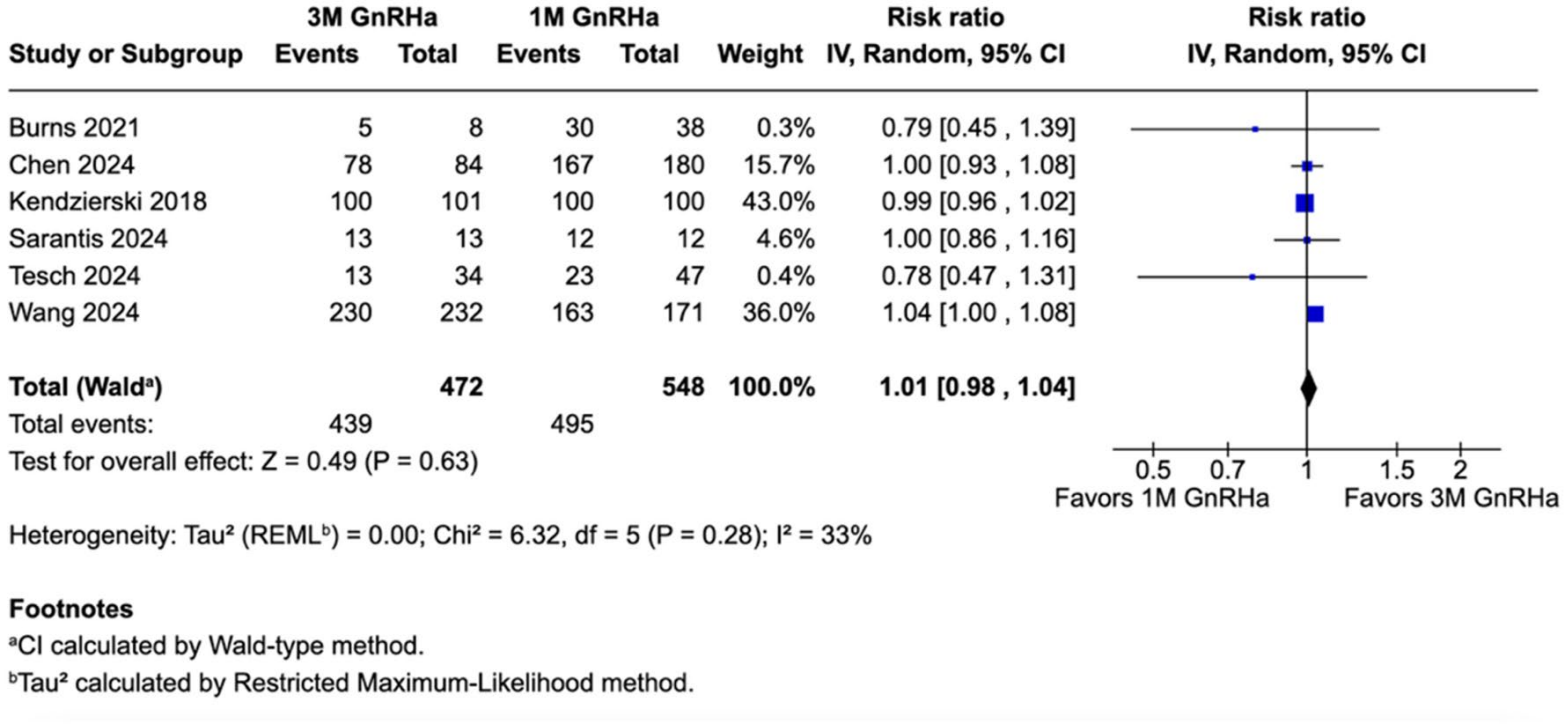
Estradiol suppression with 3 M versus 1 M GnRHa regimens, showing no significant inter-schedule differences (p = 0.63)

Estradiol suppression was reported in six studies. Pooled analyses demonstrated that both 3 M and 1 M GnRHa regimens achieved comparable rates of estradiol suppression, with no significant between-group differences (RR 1.01; 95% CI 0.98–1.04; p = 0.63; I^2^ = 33%; Fig. 2). Among these studies, follow-up duration was reported in one trial [48], with a median follow-up of 84 months.

**Fig. 3:**
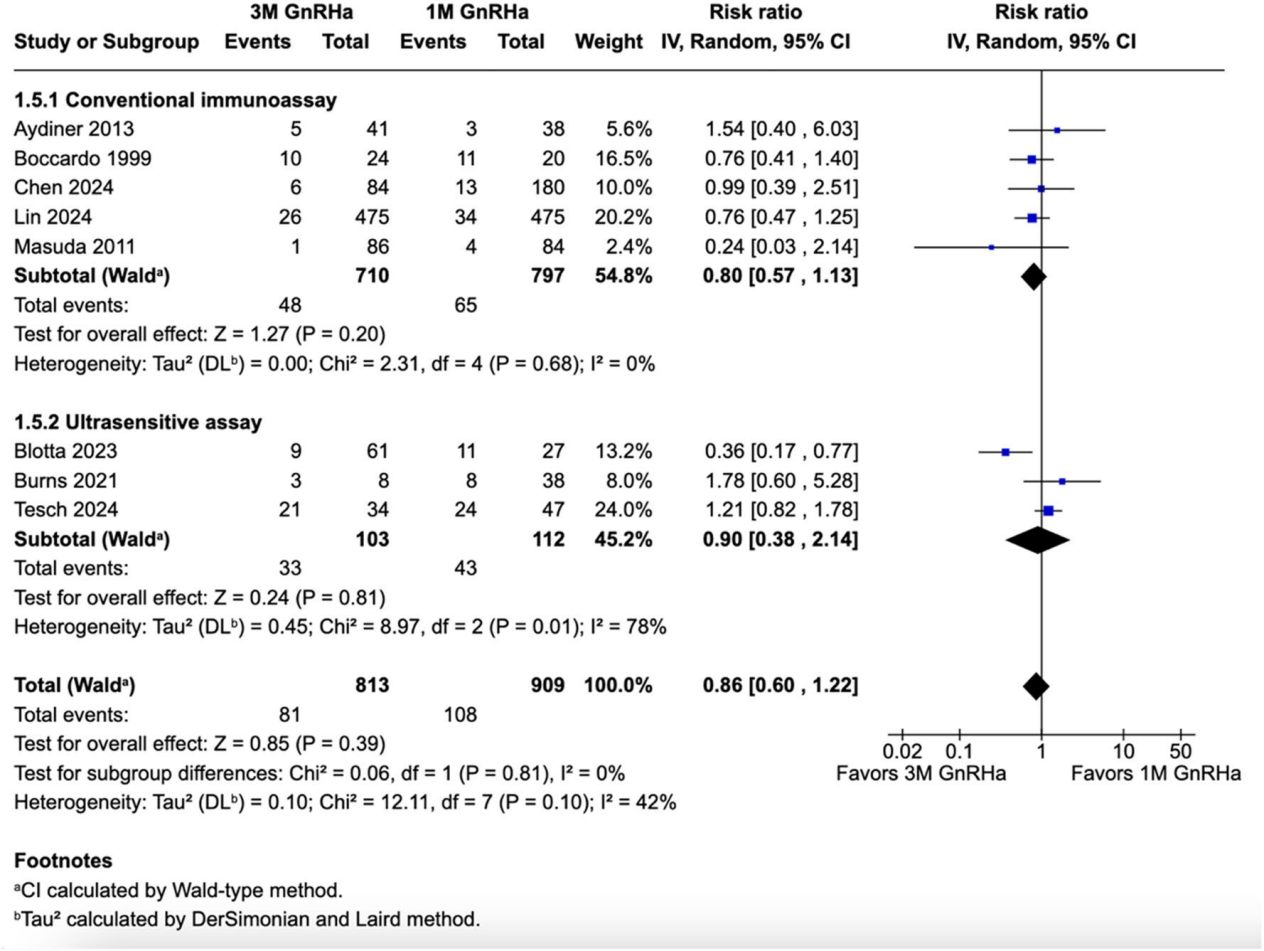
Ovarian escape with 3 M versus 1 M GnRHa regimens, showing no significant inter-schedule differences in pooled analyses (p = 0.39), irrespective of estradiol assay type (conventional immunoassay or ultrasensitive)

OE was reported in eight studies. The overall incidence of OE was 10% and did not differ significantly between schedules (RR 0.86; 95% CI 0.60–1.22; p = 0.39; I^2^ = 42%). Subgroup analyses stratified by E2 assay methodology showed consistent results (Fig. 3). In trials using ultrasensitive assays, higher absolute OE detection was reported (31%) but without significant difference between regimens (RR 0.90; 95% CI 0.38–2.14; p = 0.81; I^2^ = 78%), while those employing conventional immunoassays similarly showed no significant difference (RR 0.80; 95% CI 0.57–1.13; p = 0.20; I^2^ = 0%). Among studies reporting follow-up data, median follow-up ranged from 22.5 to 84 months.

**Fig. 4:**
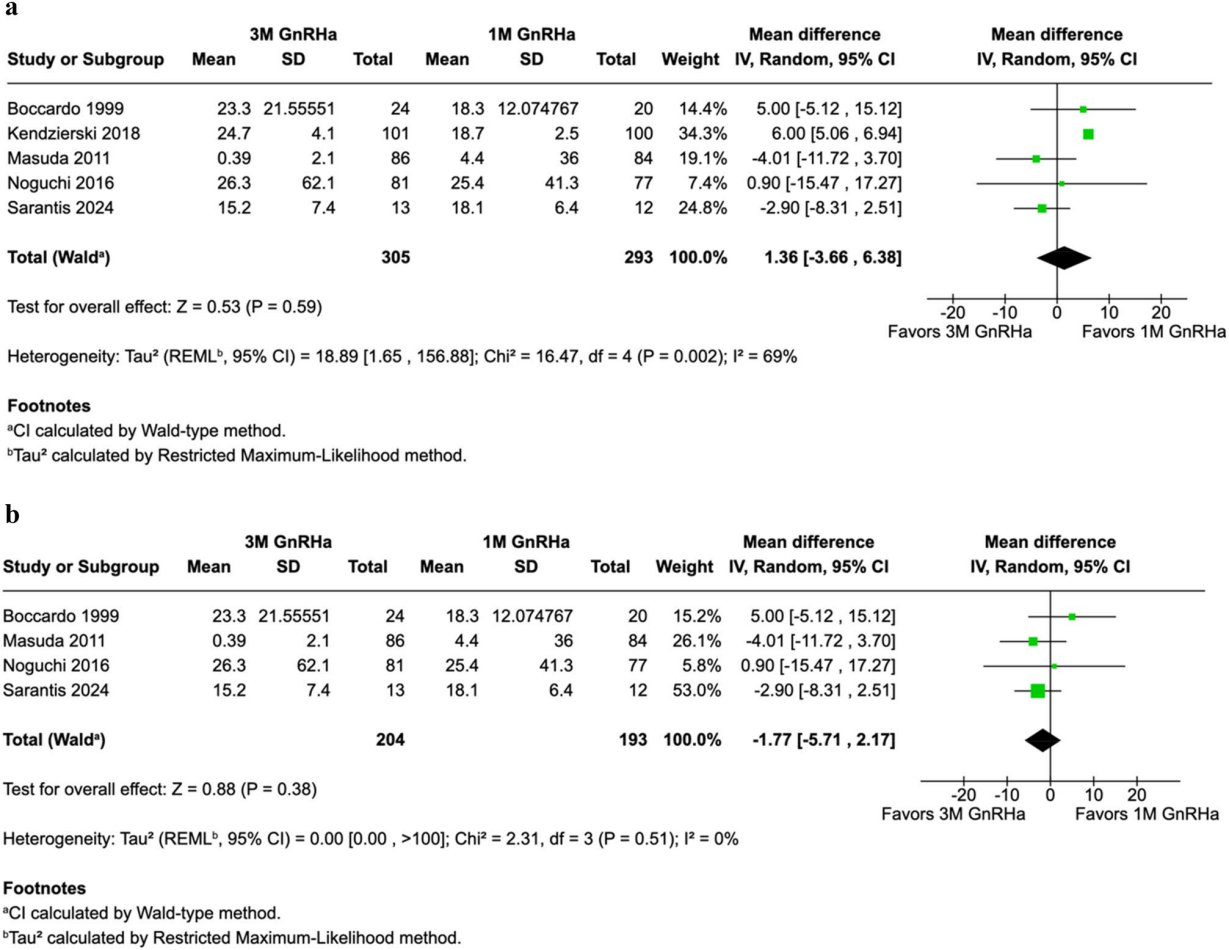
**a** Mean estradiol levels at 12 weeks with 3 M versus 1 M GnRHa regimens: overall pooled analysis. Analyses showed no significant inter-schedule differences (p = 0.59). **b** Mean estradiol levels at 12 weeks with 3 M versus 1 M GnRHa regimens: sensitivity analysis restricted to RCTs. Analyses showed no significant inter-schedule differences (p = 0.38)

Mean E2 concentrations were reported in five studies, four of which used conventional immunoassays [25, 27, 29, 31], while one did not specify the assay methodology [43]. Levels were equivalent between the two dosing schedules (MD 1.36 pg/mL; 95% CI –3.66 to 6.38; p = 0.59; I^2^ = 69%; Fig. 4a). A sensitivity analysis restricted to RCTs corroborated these findings (MD –1.77 pg/mL; 95% CI –5.71 to 2.17; p = 0.38; I^2^ = 0%; Fig. 4b). No significant differences were observed in mean FSH and LH levels (Supplementary Fig. 1 and Supplementary Fig. 2, respectively).

In the four trials that reported menstrual outcomes, complete amenorrhea was achieved in all participants within 12 to 16 weeks of follow-up. In two studies [24, 29], patients received tamoxifen concomitantly with GnRHa; in one study [26], ET consisted of either tamoxifen or AI in combination with GnRHa; and in one study [25], GnRHa was administered as endocrine monotherapy. Across these settings, no differences between 3 and 1 M regimens were observed in the achievement of amenorrhea.

### Survival outcomes

**Fig. 5:**
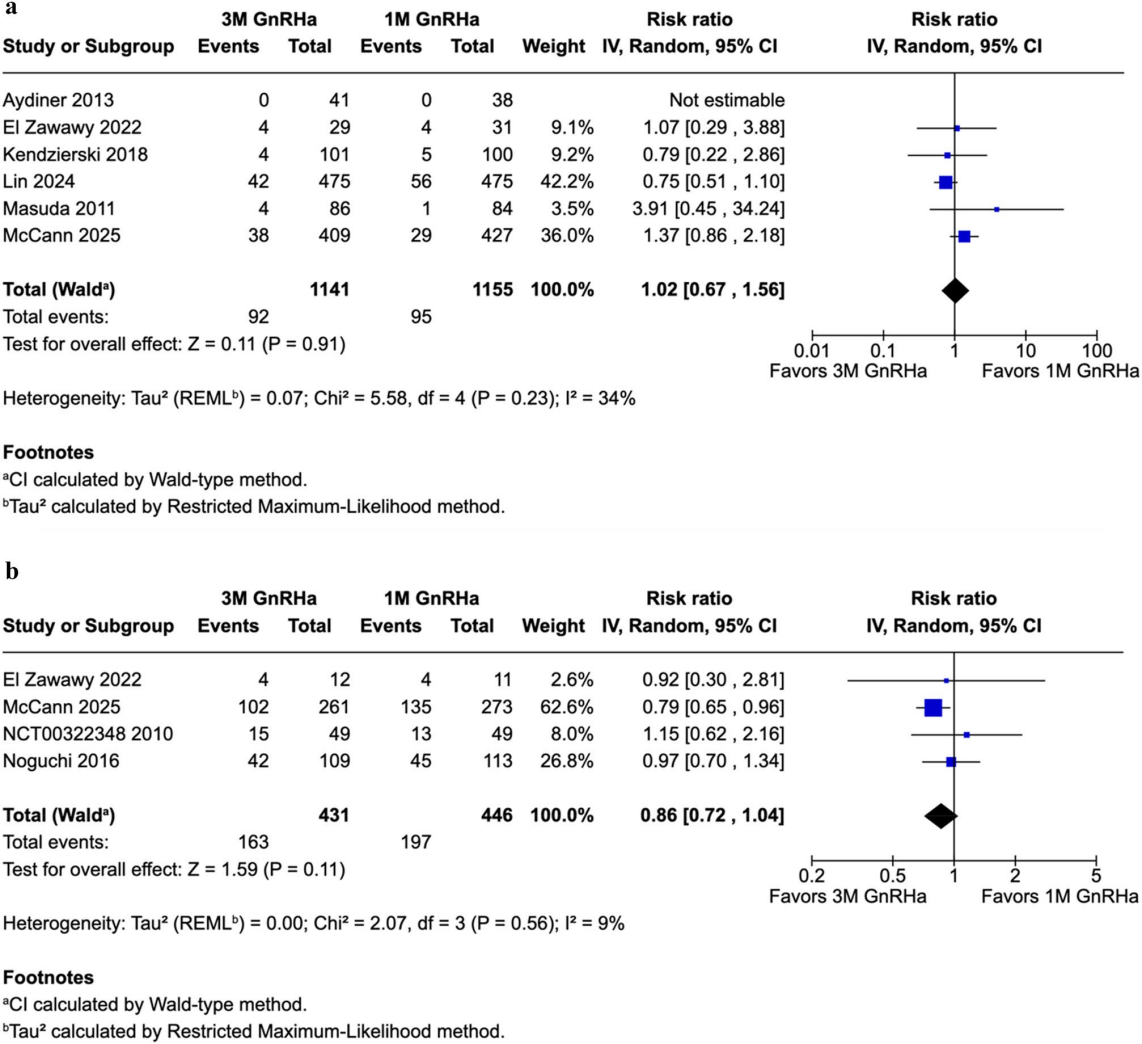

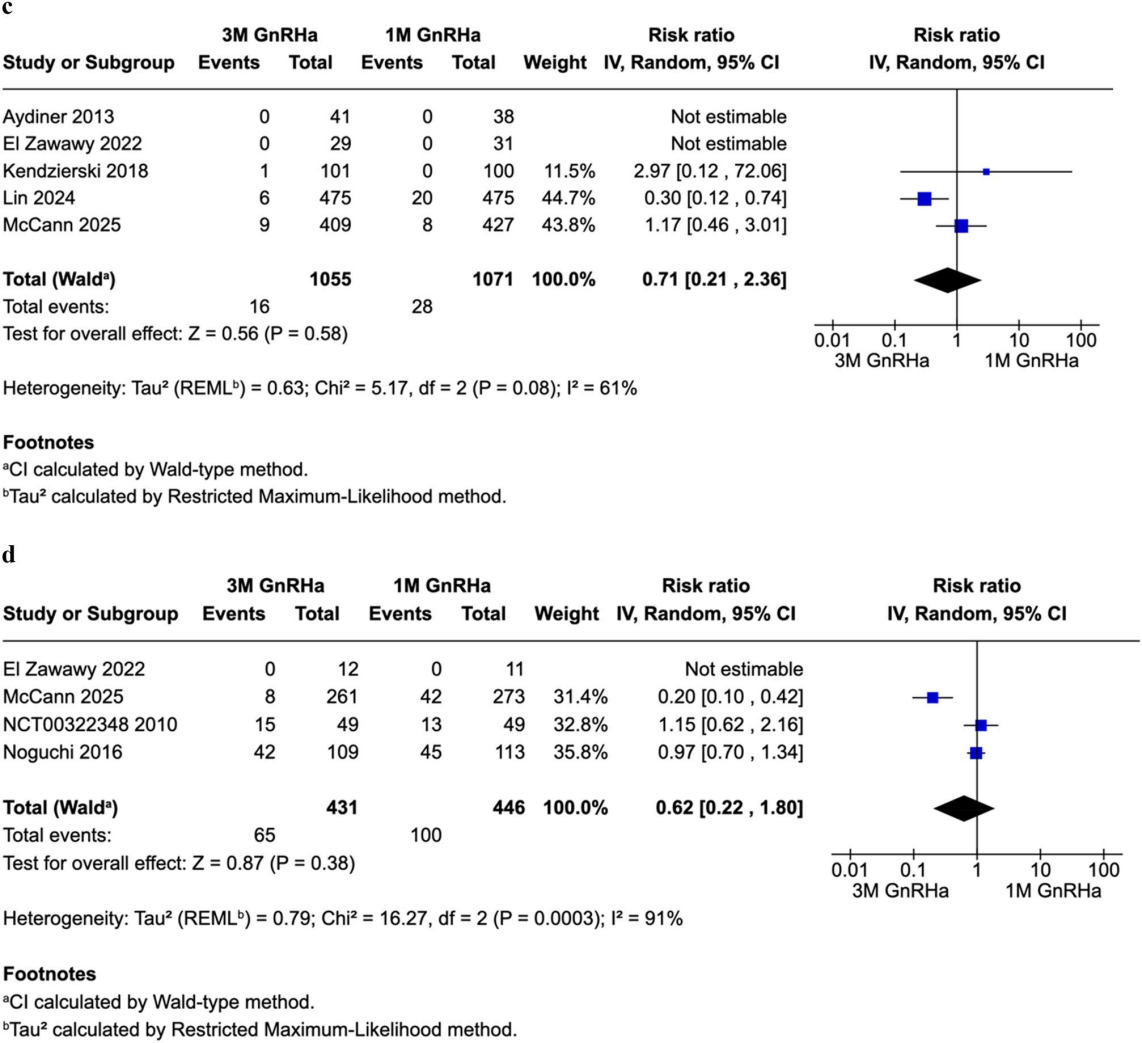
**a** Survival outcomes with 3 M versus 1 M GnRHa regimens: disease-free survival (DFS) events; No significant inter-schedule differences were observed (p = 0.91). **b** Survival outcomes with 3 M versus 1 M GnRHa regimens: progression-free survival (PFS) events; No significant inter-schedule differences were observed (p = 0.11). **c** Survival outcomes with 3 M versus 1 M GnRHa regimens: all-cause mortality in early stage disease; No significant inter-schedule differences were observed (p = 0.58). **d** Survival outcomes with 3 M versus 1 M GnRHa regimens: all-cause mortality in advanced/metastatic disease; No significant inter-schedule differences were observed (p = 0.38)

DFS was reported in six studies, including four conducted exclusively in early stage disease and two enrolling mixed populations. For mixed studies, only data corresponding to early stage (adjuvant) cohorts were extracted. Pooled analyses showed no significant differences between 3 and 1 M regimens (RR 1.02; 95% CI 0.67–1.56; p = 0.91; I^2^ = 34%; Fig. 5a). Among studies reporting follow-up data, median follow-up ranged from 21 to 96 months.

PFS was reported in four studies, including two conducted exclusively in advanced/metastatic disease and two enrolling mixed populations. In these studies, data from advanced/metastatic cohorts were used for analysis. No significant difference between dosing schedules was observed (RR 0.86; 95% CI 0.72–1.04; p = 0.11; I^2^ = 9%; Fig. 5b). Among studies reporting follow-up data, median follow-up ranged from 21 to 37 months.

Overall survival (OS) was reported in seven studies and analyzed separately according to disease stage. In early stage disease, mortality occurred in 1.5% (16/1055) of patients receiving 3 M GnRHa and 2.6% (28/1071) receiving 1 M GnRHa (RR 0.71; 95% CI 0.21–2.36; p = 0.58; I^2^ = 61%; Fig. 5c). Among studies in this subgroup that reported follow-up data, median follow-up ranged from 21 to 37 months.

In advanced/metastatic disease, mortality occurred in 15.1% (65/431) of patients in the 3 M group and 22.4% (100/446) in the 1 M group (RR 0.62; 95% CI 0.22–1.80; p = 0.38; I^2^ = 91%; Fig. 5d). Among studies reporting follow-up data, median follow-up ranged from 21 to 37 months.

### Safety outcomes

**Fig. 6:**
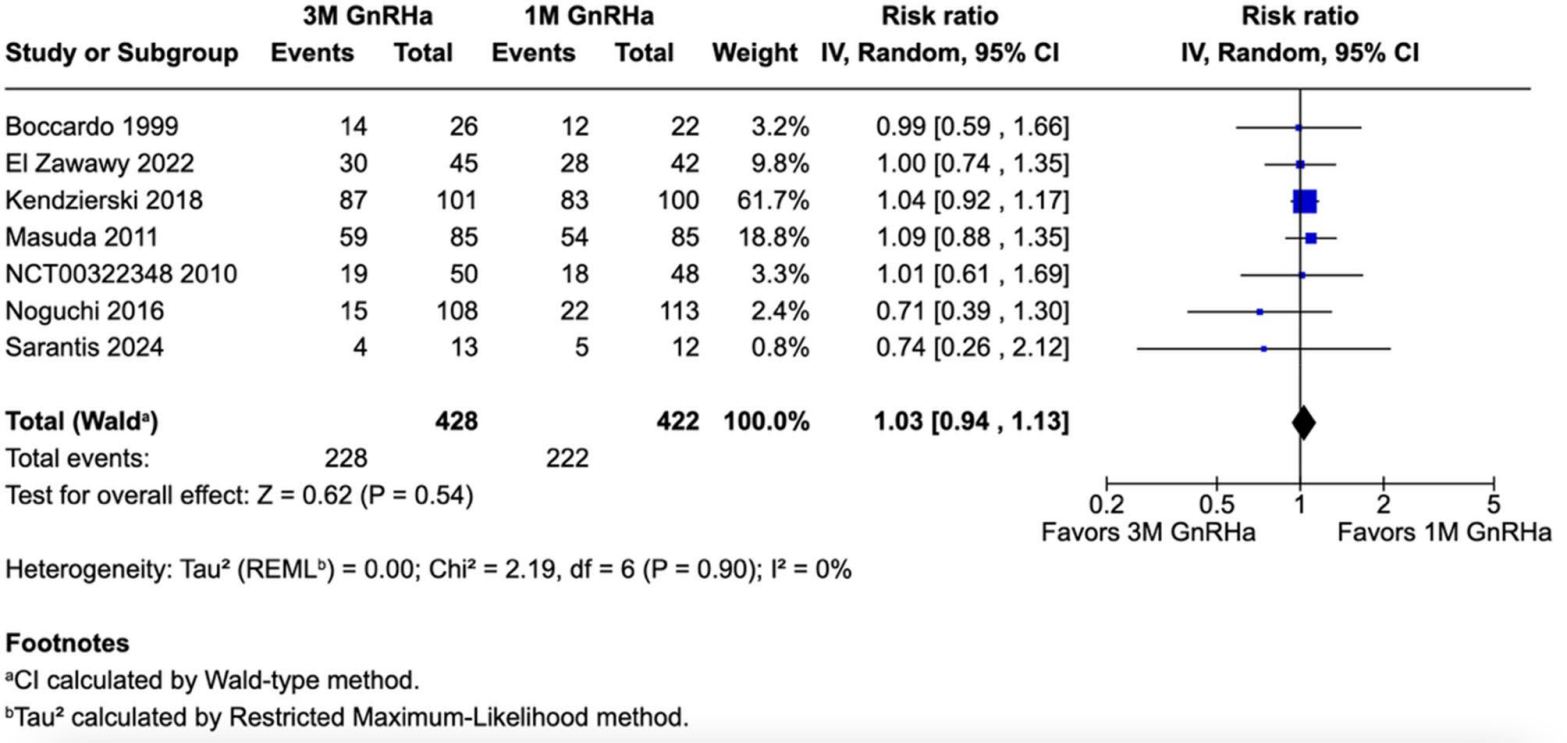
Hot flashes with 3 M versus 1 M GnRHa regimens, showing no significant inter-schedule differences in pooled analyses (p = 0.59)

Safety outcomes were not uniformly reported across studies, with hot flashes, arthralgia, headache, and nausea being the most consistently assessed adverse events. Pooled analyses showed no statistically significant differences between 3 and 1 M GnRHa regimens. Hot flashes were reported in seven studies, with similar rates between schedules (53.3% vs 52.6%; RR 1.03; 95% CI 0.94–1.13; p = 0.54; I^2^ = 0%; Fig. 6). Likewise, no significant differences were observed for arthralgia (four studies; RR 0.79; 95% CI 0.50–1.24; p = 0.30; I^2^ = 0%; Supplementary Fig. 3a), headache (six studies; RR 0.88; 95% CI 0.62–1.24; p = 0.46; I^2^ = 0%; Supplementary Fig. 3b), or nausea (five studies; RR 0.64; 95% CI 0.32–1.28; p = 0.21; I^2^ = 25%; Supplementary Fig. 3c).

### Risk of bias and certainty of evidence assessment

All RCTs were assessed as having a low risk of bias across all evaluated domains, as shown in Supplementary Fig. 4a and Supplementary Fig. 4b. In contrast, most NRSIs were judged to have a serious risk of bias, predominantly due to residual confounding, while a minority were rated as moderate risk for applying appropriate methods to account for measured confounders (Supplementary Fig. 5a and Supplementary Fig. 5b).

Assessment of publication bias was constrained by the limited number of studies available for each outcome (eight studies contributed data on OE). On visual inspection, the funnel plot for OE (Supplementary Fig. 6) demonstrated clustering of studies in the upper region of the graph, consistent with larger sample sizes and higher precision, with a relatively symmetrical distribution around the pooled effect estimate. However, the small number of contributing studies precludes reliable evaluation of asymmetry, and potential publication bias cannot be excluded.

According to the GRADE assessment, the certainty of evidence was rated as moderate for mean E2 levels, DFS events, and hot flashes, and low for OE, with downgrades mainly related to concerns regarding publication bias and indirectness (Supplementary Table 2).

## Discussion

In this systematic review and meta-analysis of 15 studies involving more than 4,000 patients, the main findings were: (1) comparable rates of estradiol suppression and similar mean concentrations of E2, FSH, and LH at 12 weeks between 3 and 1 M regimens; (2) higher absolute detection of OE when ultrasensitive assays were used, without differences between dosing schedules; (3) no statistically significant differences in survival outcomes, including DFS, PFS or all-cause mortality; and (4) a similar toxicity profile between regimens.

Given the well-established benefit of reversible OFS with GnRHa in premenopausal women with high-risk BC [14, 49], and the current availability of 3 M formulations that substantially reduce the number of injections and healthcare visits, there is increasing clinical interest in determining whether this more practical schedule is as effective as the 1 M regimen. Over the past two decades, evidence from randomized trials [25, 29, 31, 43, 44] and observational cohorts [24, 26–28, 30, 32, 45–48] has accumulated, supporting the non-inferiority of the 3 M approach in achieving OFS. More recently, driven in part by the COVID-19 pandemic, the growing body of data has prompted updates in clinical guidelines and regulatory approvals [33–36], although its use is not yet universally implemented across clinical settings.

Despite mounting evidence of therapeutic equivalence, some hesitation persists within the oncology community regarding the widespread adoption of the extended-interval formulations [50]. Most individual studies included in this field are relatively small, focused primarily on measures of OFS effectiveness, using heterogeneous parameters, and only a minority include robust survival analyses. Many of them lack sufficient statistical power to provide a definitive answer. Nevertheless, despite variability in study design, patient populations, interventions, estradiol assays, and reported outcomes, the collective body of evidence—and our meta-analysis—supports the equivalence of the 3 M and 1 M formulations.

The ability to sustain complete ovarian suppression over time has become an area of particular focus, given its potential impact on ET effectiveness. OE may manifest as either transient elevations or persistent recovery of premenopausal E2 levels during GnRHa therapy. In the present meta-analysis, OE was defined according to study-specific criteria and was considered present when at least one E2 measurement exceeded the menopausal threshold established by the individual trial. Importantly, the clinical significance of OE remains a matter of debate. While persistent biochemical recovery could theoretically attenuate the therapeutic benefit of OFS, transient elevations detected on isolated measurements do not necessarily translate into clinically meaningful loss of ovarian suppression or worse oncologic outcomes [51–54]. Within the pooled dataset, rates of OE were comparable between the 3 M and 1 M regimens.

Although recommendations for hormonal monitoring during OFS remain inconsistent [55], accumulating evidence indicates that OE detection is strongly influenced by the assay method, with ultrasensitive assays identifying substantially higher rates [56]. Notably, only three studies included in the present analysis employed ultrasensitive E2 assays. Overall, the incidence of OE was approximately 10%, but reached 31% among studies using ultrasensitive assays, comparable to the 17% per-timepoint OE rate reported in the SOFT-EST substudy [57]. The slightly higher incidence observed in our ultrasensitive subgroup may be partly explained by the inclusion of younger patients in one study [50], as younger age is associated with greater ovarian reserve and a higher likelihood of incomplete suppression [56].

The clinical implications of OE may also differ according to the ET backbone. AIs require complete ovarian suppression to maintain a postmenopausal hormonal environment, as residual ovarian activity may lead to compensatory gonadotropin stimulation and inadequate estrogen deprivation. In contrast, tamoxifen exerts its antitumor effect through estrogen receptor modulation and retains activity even in the presence of circulating estradiol. Consequently, incomplete or transient ovarian suppression may be of greater theoretical concern in patients receiving AI-based regimens than in those treated with tamoxifen [58]. However, the available data did not permit stratified analyses according to type of ET or correlation of biochemical OE with recurrence or survival outcomes at the individual level, precluding definitive conclusions regarding its clinical impact.

Amenorrhea represents another commonly reported clinical surrogate of ovarian suppression, although its interpretation varies according to the ET context [57]. In the trials reporting menstrual outcomes, complete amenorrhea was consistently achieved across ET backbones, including tamoxifen, AIs, and GnRHa monotherapy, with no differences between dosing schedules. These findings are further supported by the absence of significant differences in mean E2, FSH and LH concentrations between regimens, reinforcing the overall consistency of hormonal suppression observed in this analysis.

Treatment adherence represents another clinically relevant dimension in HR + BC, as nonadherence and early discontinuation of therapy are consistently associated with worse survival outcomes [59]. Beyond the adverse effects of ET that may compromise quality of life [60], GnRHa use also introduces practical burdens, including injection-site pain, local reactions, and the need for regular visits to a healthcare facility for administration [61]. Evidence from prostate cancer suggests that longer-acting formulations (3-month and 6-month) are associated with greater patient satisfaction, reduced anxiety, and an increased sense of autonomy [62], which may ultimately improve compliance [63]. From a cost-effectiveness standpoint, studies in prostate cancer also indicate that extended-interval formulations appear more favorable, with 3 M regimens costing at least 40% less than 1 M injections, largely due to reduced visit and monitoring requirements [64]. Such advantages could simultaneously improve patient experience, reinforce long-term adherence, and generate meaningful resource savings for healthcare systems.

While the current evidence supports therapeutic equivalence, further prospective investigations are warranted. Future studies should incorporate standardized ultrasensitive estradiol monitoring with predefined definitions of OE and longitudinal hormonal assessments. Importantly, the clinical relevance of OE should be examined in relation to oncologic outcomes, including recurrence and survival events. In addition, the integration of patient-reported outcomes, including quality of life and adherence measures, as well as formal cost-effectiveness analyses conducted in breast cancer populations, will be important to further inform the optimal implementation of extended-interval GnRHa strategies.

Our study has several limitations. First, the small number of RCTs assessing comparable endpoints precluded conducting RCT-only meta-analyses for most outcomes. To mitigate potential design-related imbalances, we performed a sensitivity analysis restricted to RCTs for mean estradiol levels, which yielded results consistent with the overall dataset including NRSIs. Second, several NRSIs were judged to have serious risk of bias, primarily related to confounding and participant selection. Residual confounding, selection bias, and chance findings cannot be excluded. Third, survival outcomes require cautious interpretation. Overall survival was characterized by substantial heterogeneity and a limited number of events. Follow-up duration was inconsistently reported and frequently short for time-dependent endpoints such as DFS and OS. Accordingly, survival findings, especially OS, should be considered exploratory. Fourth, heterogeneity in definitions and measurement of hormone-related endpoints represents an additional limitation. Although we performed subgroup analyses according to estradiol assay type, only three studies used ultrasensitive assays. Moreover, we were unable to evaluate OE according to assay methodology in conjunction with ET backbone, nor to correlate biochemical OE with individual recurrence or survival outcomes. Fifth, available data did not permit stratified analyses according to type of ET (tamoxifen versus AIs), either for hormonal endpoints or menstrual outcomes. This limits inference regarding potential differential clinical implications of incomplete ovarian suppression across treatment backbones. Finally, hormonal outcomes were generally assessed over relatively short intervals with limited serial measurements. The absence of standardized longitudinal monitoring may have underestimated the true incidence and temporal patterns of OE.

## Conclusion

In summary, the 3 M administration of GnRHa in premenopausal women with HR + BC achieves OFS comparable to 1 M dosing, without compromising survival outcomes and with a similar and expected safety profile. These findings support current regulatory approvals and guideline recommendations permitting the use of 3 M formulations in appropriately selected patients. Broader adoption of this regimen, when clinically appropriate, may further enhance treatment adherence, reduce patient burden, and improve the real-world delivery of ET.

## Supplementary Information

Below is the link to the electronic supplementary material.

Supplementary file1 (DOCX 1769 KB)

## Data Availability

All data generated during and/or analyzed during the current study are available in the current publication.

## Abbreviations

ASCO: American society of clinical oncology
BC: Breast cancer
CENTRAL: Cochrane central register of controlled trials
CI: Confidence interval(s)
DFS: Disease-free survival
ET: Endocrine therapy
E2: Estradiol
FSH: Follicle-stimulating hormone
GnRH: Gonadotropin-releasing hormone
GnRHa: Gonadotropin-releasing hormone agonist
GRADE: Grading of recommendations, assessment, development and evaluation
HR +: Hormone receptor-positive
IA: Conventional immunoassay
LH: Luteinizing hormone
MD: Mean difference(s)
NRSI: Non-randomized studies of intervention(s)
OE: Ovarian escape
OFS: Ovarian function suppression
PFS: Progression-free survival
PRISMA: Preferred reporting items for systematic reviews and meta-analysis
RCT: Randomized controlled trial(s)
RR: Risk ratio(s)
UA: Ultrasensitive assay
