# Supplementary material for: Three-monthly gonadotropin-releasing hormone agonist for ovarian function suppression in premenopausal breast cancer: a systematic review and meta-analysis
Source: Breast Cancer Res Treat. 2026 Jun 12;217(2):48. doi: 10.1007/s10549-026-07960-2 (PMC13263206; doi:10.1007/s10549-026-07960-2)
Supplement: Supplementary file 1 — Supplementary file1 (DOCX 1769 KB) [file 10549_2026_7960_MOESM1_ESM.docx]

**Supplementary Material**

**Three-Monthly Gonadotropin-Releasing Hormone Agonist for Ovarian Function Suppression in Premenopausal Breast Cancer: A Systematic Review and Meta-Analysis**

Caio Dabbous de Liz, M.D.,^#^ Hospital Paraná, Rede Américas, Maringá, (Paraná), Brazil

Pedro C. Abrahão Reis,^#^ Medical Student; Universidade Federal do Rio de Janeiro, Rio de Janeiro, Brazil

Ellen R. Blanchard-Cavagis, M.D., Université Paris Cité, Paris, France

Isabela Diniz, Medical Student, Universidade Estadual de Campinas (UNICAMP), Campinas, (São Paulo), Brazil

Heloísa Brito, Medical Student, Universidade Federal da Paraíba (UFPB), João Pessoa, (Paraíba), Brazil

Filipe Luis Vasconcelos Visani, M.D., Oncoclínicas, Salvador, (Bahia), Brazil

^#^ contributed equally

**Journal: Breast Cancer Research and Treatment**

**Address for correspondence:**

**Email**: pedroreis@ufrj.br

**Table of Contents**

[**Supplementary Table1.** Search strategy 3](#_Toc215176516)

[**Supplementary Fig1.** Mean FSH level between 3M and 1M schedule 4](#_Toc215176524)

[**Supplementary Fig2.** Mean LH level between 3M and 1M schedule 5](#_Toc215176525)

[**Supplementary Fig3.a -** Arthralgia incidence between 3M and 1M schedule 6](#_Toc215176526)

[**Supplementary Fig3.b -** Headache incidence between 3M and 1M schedule 6](#_Toc215176527)

[**Supplementary Fig3.c -** Nausea incidence between 3M and 1M schedule 6](#_Toc215176528)

[**Supplementary Fig4a.** Risk of bias in randomized trials (RoB2) 7](#_Toc215176529)

[**Supplementary Fig4b.** Summary of Rob2 7](#_Toc215176530)

[**Supplementary Fig5a.** Risk of bias in non-randomized trials (ROBINS-I) 9](#_Toc215176531)

[**Supplementary Fig5b.** Summary of ROBINS-I 9](#_Toc215176532)

[**Supplementary Fig6.** Publication bias assessment by funnel plot. 10](#_Toc215176533)

[**Supplementary Table2.** GRADE assessment 11](#_Toc215176534)

# **Supplementary Table1 -** Search strategy

| **Databases** | **Search Strategy** | **Number of results** |
| --- | --- | --- |
| **PubMed** | (goserelin OR leuprolide OR triptorelin OR "GnRH agonist" OR "GnRH agonists" OR "LHRH agonist" OR "LHRH agonists") AND ("long-acting" OR "3 month" OR "3 months" OR "3-monthly" OR trimonthly OR "every 3-month" OR "12 weeks") AND ("every month" OR monthly OR "1-month" OR "4 weeks") AND "breast cancer" | 25 results |
| **Embase** |  | 82 results |
| **Cochrane** |  | 97 results |

# **Supplementary Fig1.** Mean follicle-stimulating hormone (FSH) levels at 12 weeks with 3M versus 1M GnRHa regimens. Analyses showed no significant inter-schedule differences (p=0.17)


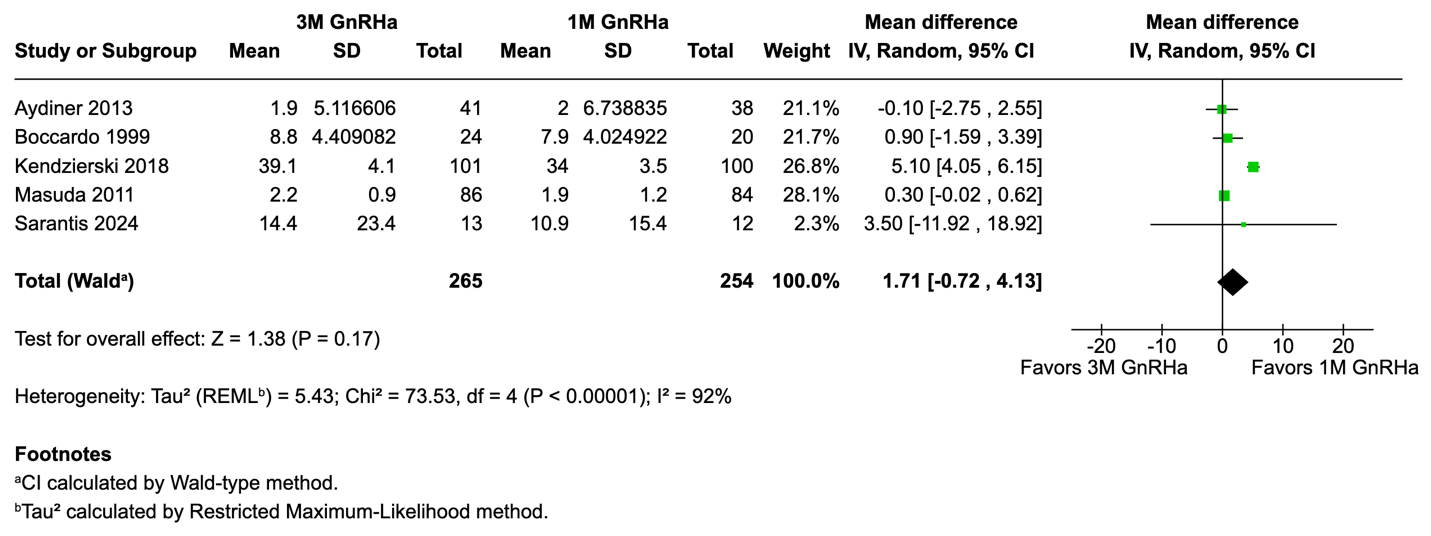


# **Supplementary Fig2.** Mean luteinizing hormone (LH) levels at 12 weeks with 3M versus 1M GnRHa regimens. Analyses showed no significant inter-schedule differences (p=0.09)


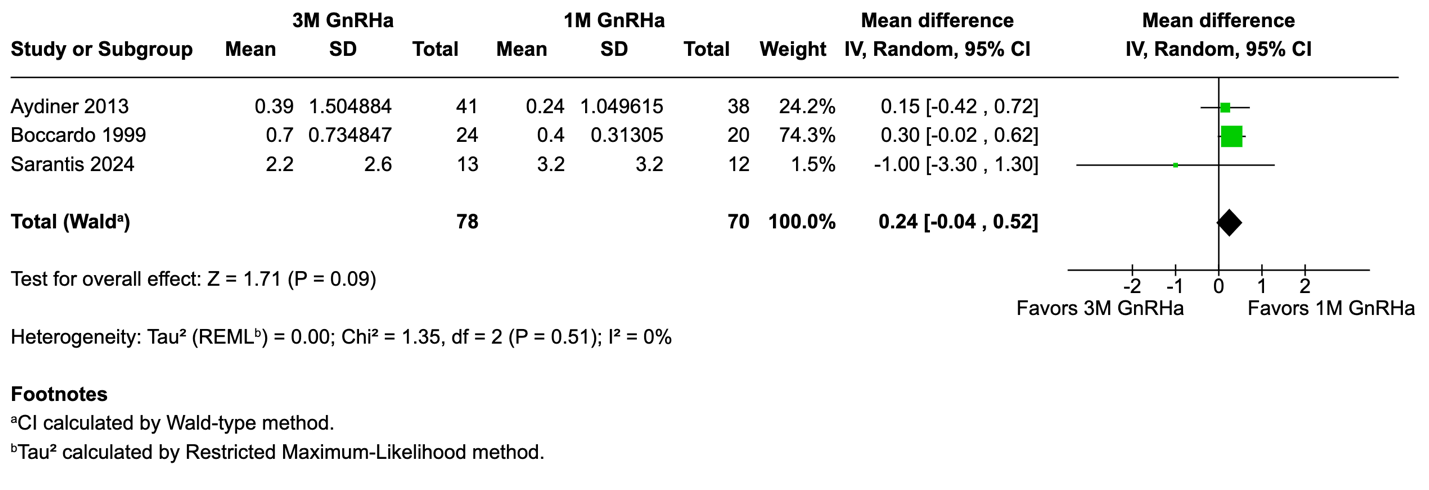


# **Supplementary Fig3.a -** Safety outcomes with 3M versus 1M GnRHa regimens: arthralgia. No significant inter-schedule differences were observed across pooled analyses (p=0.30)


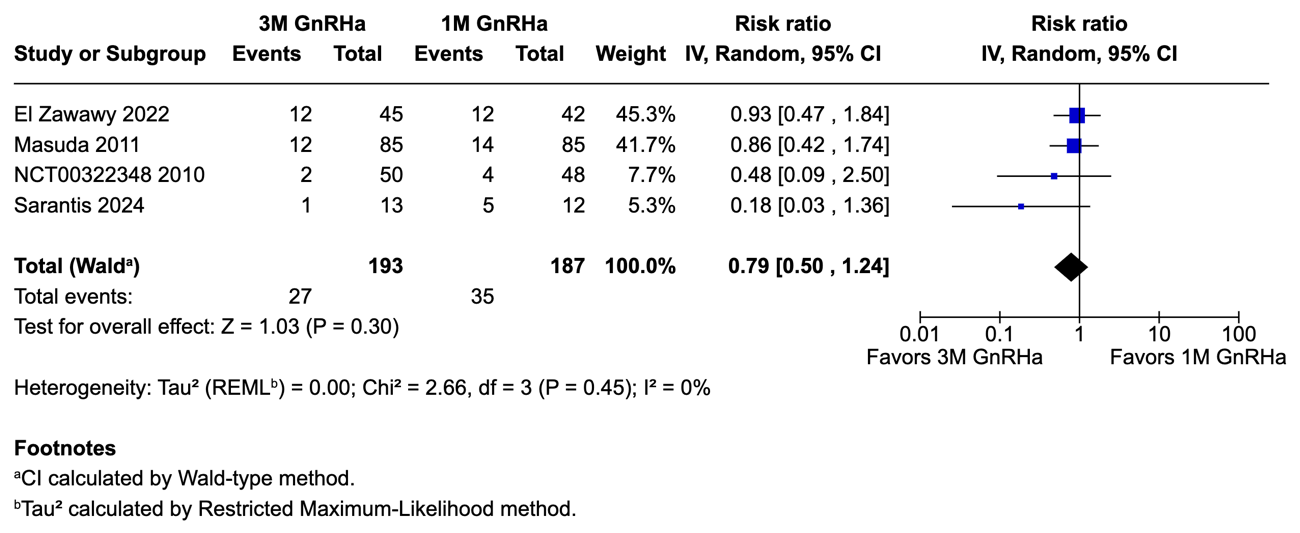


**Supplementary Fig3.b -** Safety outcomes with 3M versus 1M GnRHa regimens: headache. No significant inter-schedule differences were observed across pooled analyses (p=0,46)


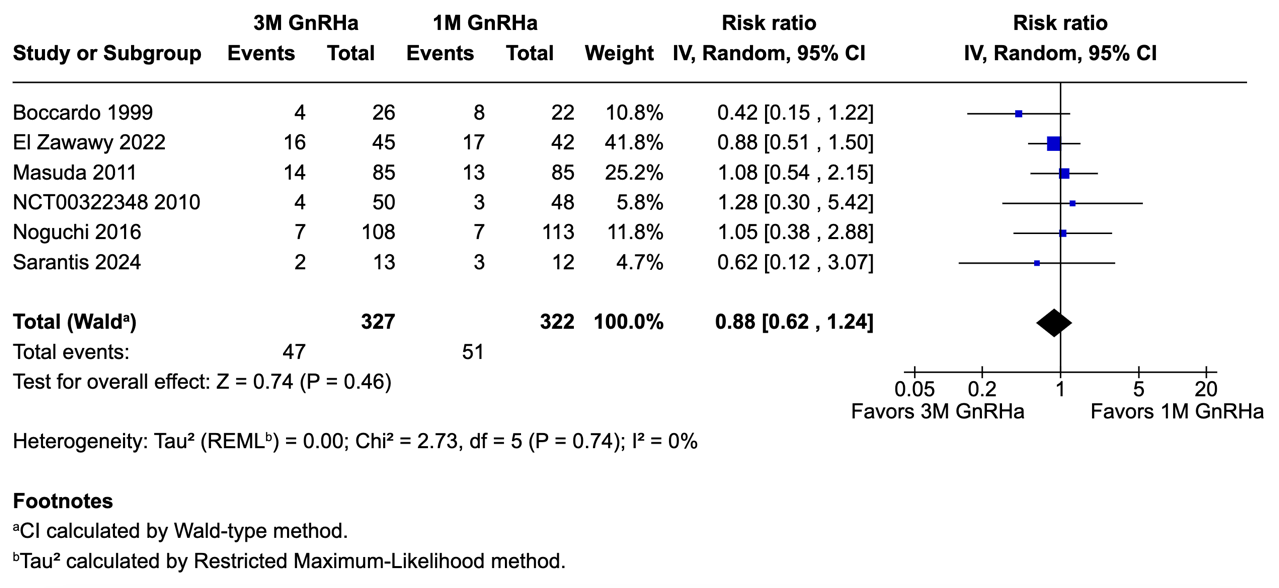


**Supplementary Fig3.c -** Safety outcomes with 3M versus 1M GnRHa regimens: nausea. No significant inter-schedule differences were observed across pooled analyses (p=0,21)


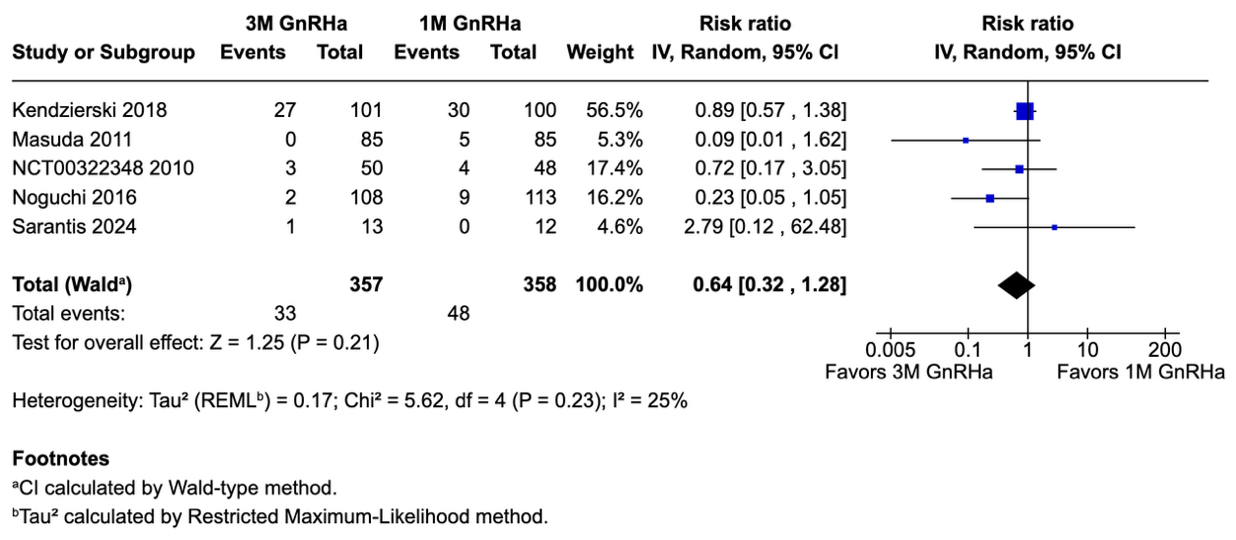


**Supplementary Fig4a.** Critical appraisal of individual studies according to the Cochrane Collaboration’s tool for assessing risk of bias in randomized trials (RoB2).


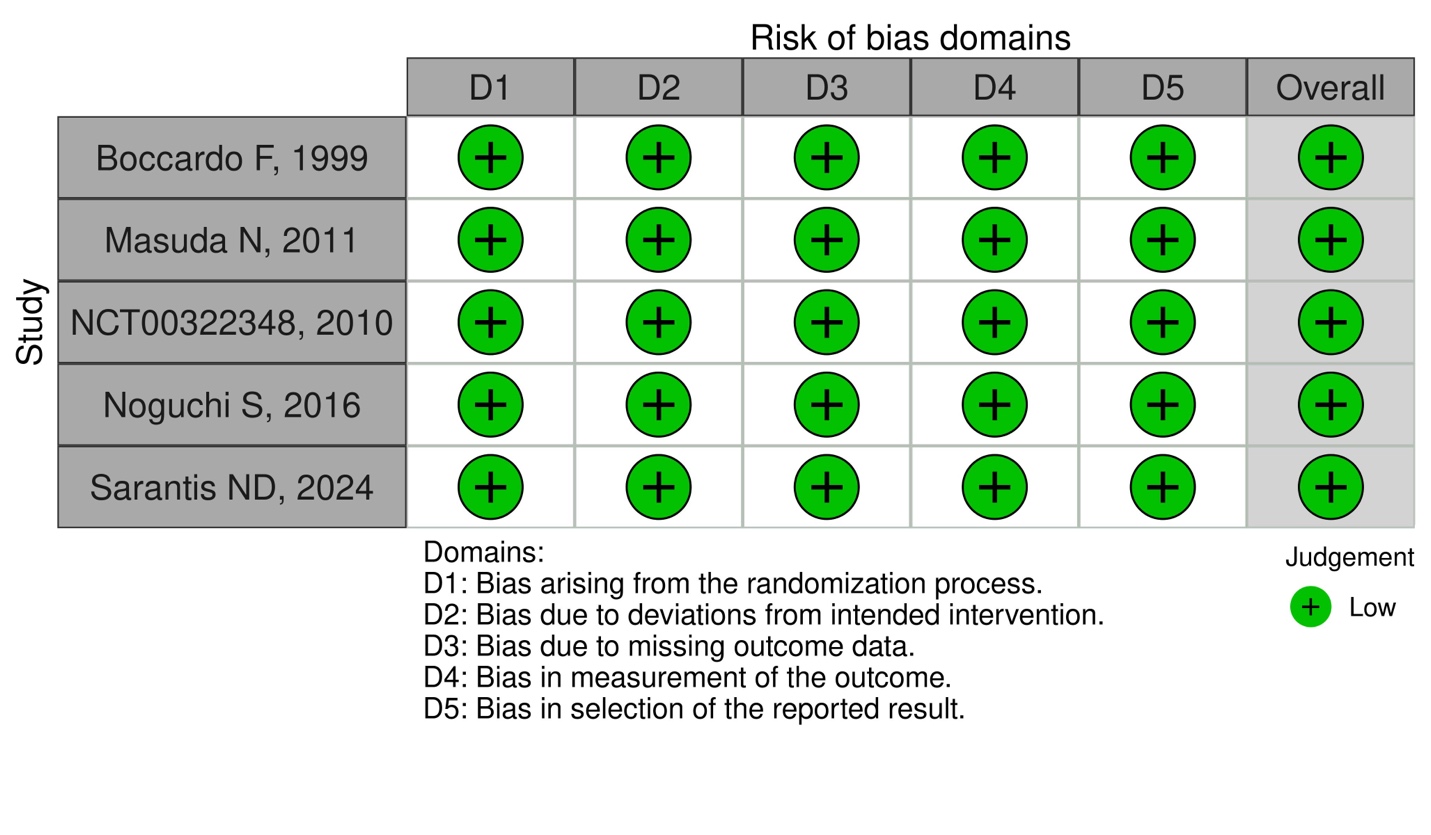


**Supplementary Fig4b.** Summary of Rob2. All included RCTs were judged to be at low risk of bias across all assessed domains.

**
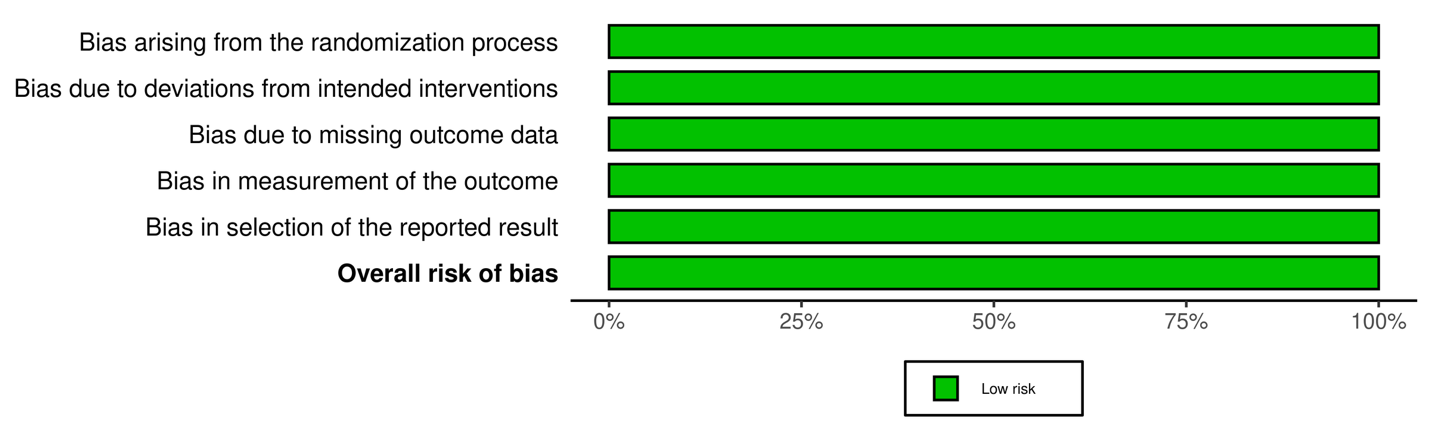
**

# **Supplementary Fig5a.** Critical appraisal of individual studies according to the Cochrane Collaboration’s tool for assessing risk of bias in non-randomized trials (ROBINS-I)

**
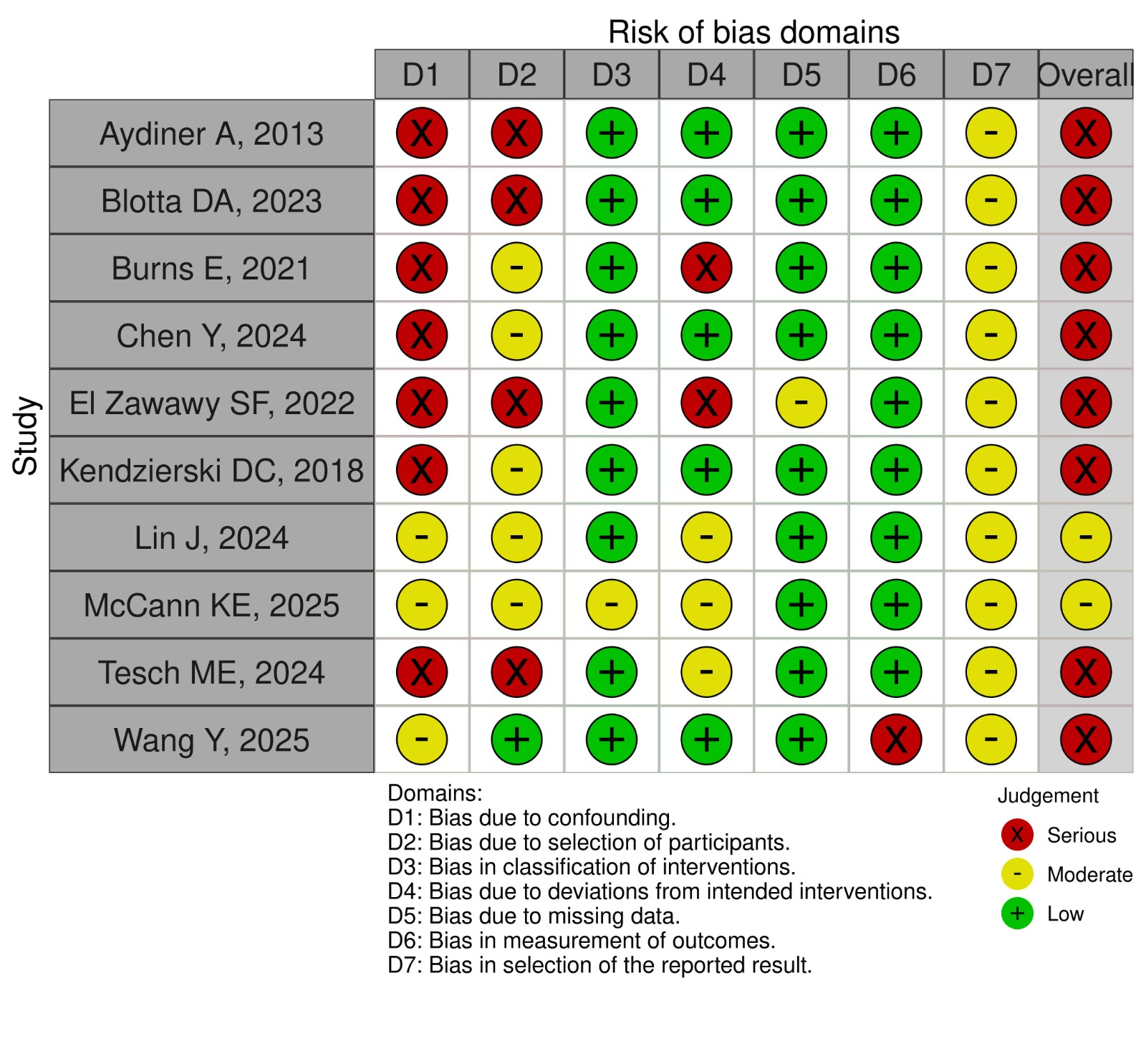
**

**Supplementary Fig5b.** Summary of ROBINS-I. Most NRSIs were judged to have a serious risk of bias, predominantly due to residual confounding and selection of participants, while a minority were rated as moderate risk, having applied appropriate methodological strategies to mitigate potential confounders.

**
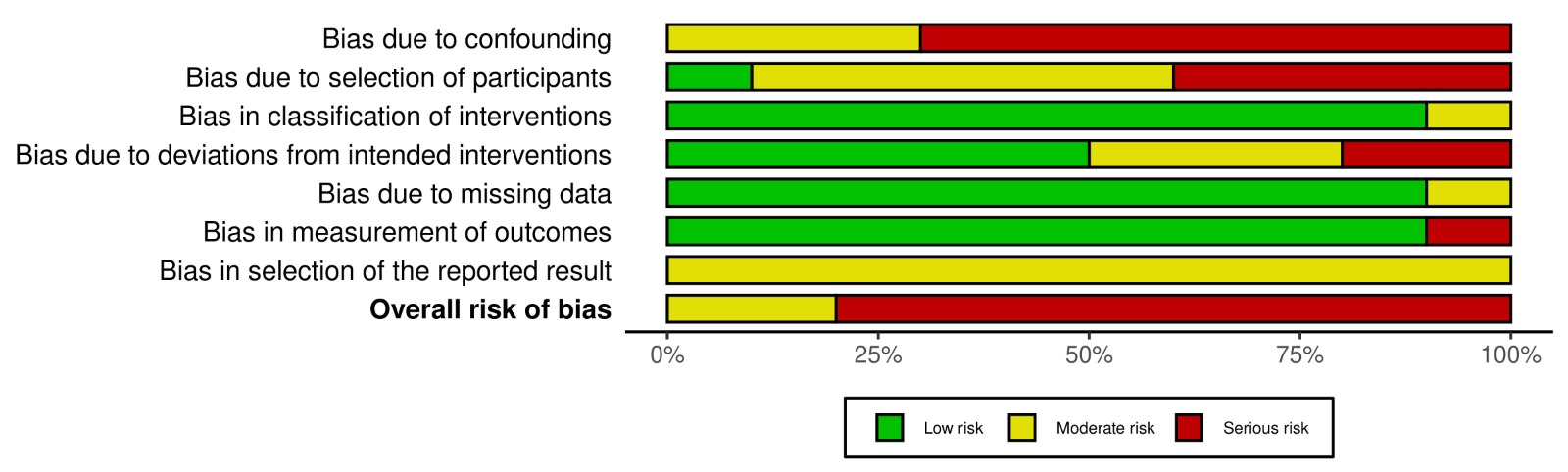
**

**Supplementary Fig6.** Funnel plot for the outcome of ovarian escape. Visual inspection showed approximate symmetry, suggesting no clear evidence of publication bias, though the limited number of included studies precludes definitive assessment.


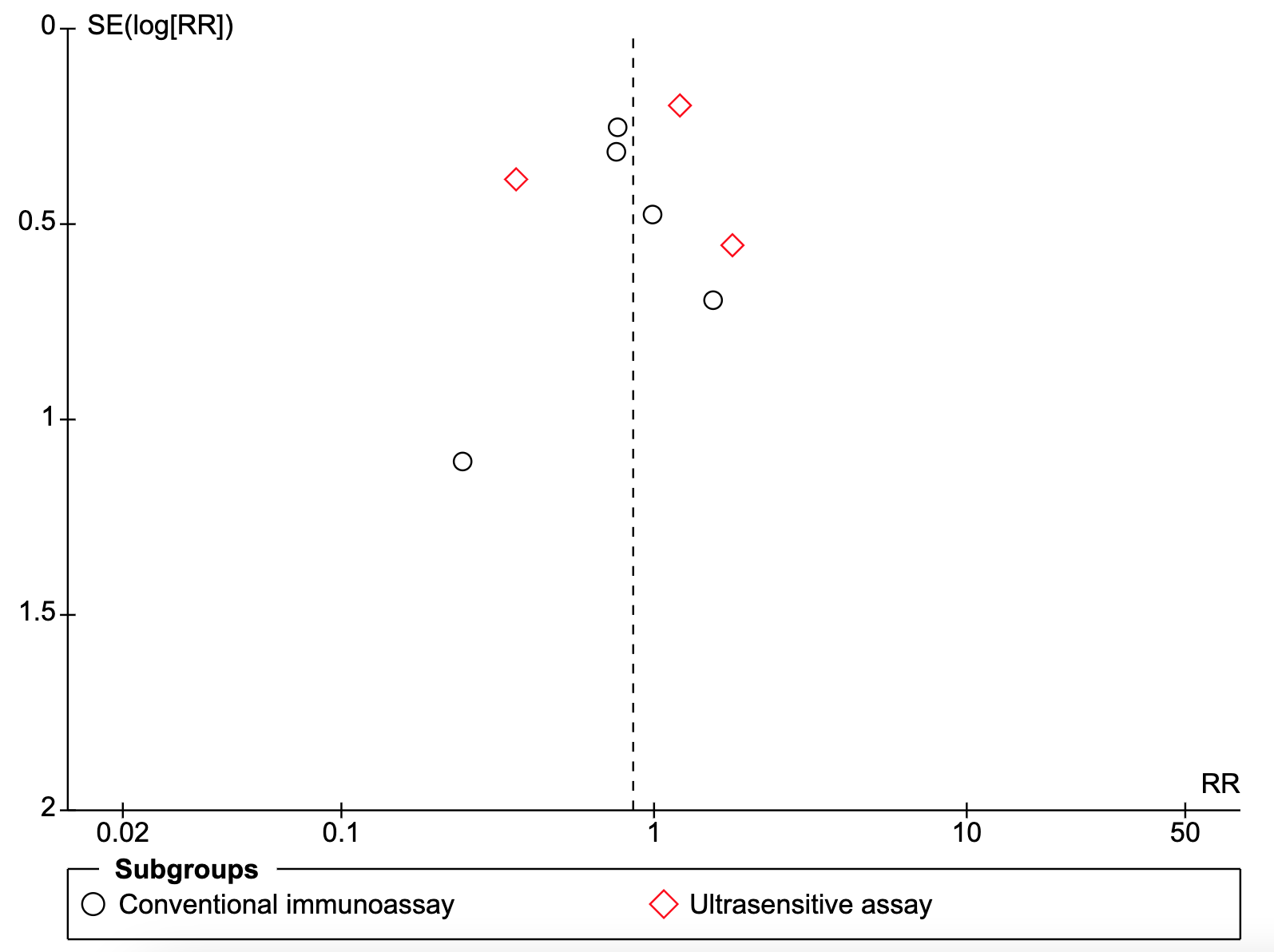


**Supplementary Table2.** The Grading of Recommendations, Assessment, Development, and Evaluations (GRADE) assessment of the certainty of evidence for key outcomes. Certainty was rated as moderate for mean E2 levels, DFS events, and hot flashes, and low for ovarian escape, with downgrades mainly due to concerns regarding publication bias and indirectness.

| **3M GnRHa compared to 1M GnRHa for OFS in premenopausal women with HR+ BC** | | | | | |
| --- | --- | --- | --- | --- | --- |
| **Patient or population:** Premenopausal women with HR+ BC  **Setting:** OFS  **Intervention:** 3M GnRHa  **Comparison:** 1M GnRHa | | | | | |
| **Outcomes** | **№ of participants (studies) Follow-up** | **Certainty of the evidence (GRADE)** | **Relative effect (95% CI)** | **Anticipated absolute effects** | |
|  |  |  |  | **Risk with 1M GnRHa** | **Risk difference with 3M GnRHa** |
| Estradiol level at 12 weeks (E2 12 weeks) assessed with: mean  follow-up: 12 weeks | 598 (5 RCTs) | ⨁⨁⨁◯ Moderate^a^ | - | The mean estradiol level at 12 weeks ranged from **4.4 to 25.4** pg/mL | MD **1.36 pg/mL higher** (3.66 lower to 6.38 higher) |
| Ovarian Escape (OE) assessed with: number of events follow-up: range 21 months to 84 months | 1722 (8 non-randomised studies) | ⨁⨁◯◯ Low^a,b^ | **RR 0.86** (0.60 to 1.22) | 119 per 1.000 | **17 fewer per 1.000** (48 fewer to 26 more) |
| Disease-free survival events (DFS events) assessed with: number of events follow-up: range 21 months to 84 months | 2306 (6 non-randomised studies) | ⨁⨁⨁◯ Moderate^a^ | **RR 1.02** (0.68 to 1.54) | 83 per 1.000 | **2 more per 1.000** (26 fewer to 45 more) |
| Hot Flashes assessed with: number of events follow-up: range 21 months to 84 months | 850 (7 RCTs) | ⨁⨁⨁◯ Moderate^a^ | **RR 1.03** (0.94 to 1.13) | 526 per 1.000 | **16 more per 1.000** (32 fewer to 68 more) |
| ***The risk in the intervention group** (and its 95% confidence interval) is based on the assumed risk in the comparison group and the **relative effect** of the intervention (and its 95% CI).  **1M:** monthly; **3M:** 3-monthly; **CI:** confidence interval; **GnRHa:** gonadotropin release-hormone agonist; **MD:** mean difference; **OFS:** ovarian function suppression; **RR:** risk ratio. | | | | | |
| **GRADE Working Group grades of evidence** **High certainty:** we are very confident that the true effect lies close to that of the estimate of the effect. **Moderate certainty:** we are moderately confident in the effect estimate: the true effect is likely to be close to the estimate of the effect, but there is a possibility that it is substantially different. **Low certainty:** our confidence in the effect estimate is limited: the true effect may be substantially different from the estimate of the effect. **Very low certainty:** we have very little confidence in the effect estimate: the true effect is likely to be substantially different from the estimate of effect. | | | | | |

#### Explanations

a. Publication bias: −1 (many studies did not report this outcome).

b. Indirectness: -1 (short-term outcome).
